# MicroRNA-1202 plays a vital role in osteoarthritis via KCNQ1OT1 has-miR-1202-ETS1 regulatory pathway

**DOI:** 10.1186/s13018-020-01655-0

**Published:** 2020-04-06

**Authors:** Changzeng Liu, Jianming Gao, Guangyan Su, Yang Xiang, Le Wan

**Affiliations:** Department of Orthopaedics, No. 904th Hospital of the Joint Logistics Support Force of PLA, Liangxi District, Wuxi, 214044 Jiangsu China

**Keywords:** Osteoarthritis, Differentially expressed miRNAs, Function and pathway analysis, Protein-protein interaction network, Competing endogenous RNAs

## Abstract

**Background:**

This study aimed to explore the molecular mechanism of osteoarthritis (OA) and provide information about new genes as potential targets for OA treatment.

**Methods:**

Gene expression profile of GSE105027, including 12 OA serum samples (OA group) and 12 healthy serum samples (ctrl group), was downloaded. The differentially expressed miRNAs (DEMs) as well as miRNA-mRNAs interactions were investigated, followed by function and pathway investigation. Then the protein-protein interaction (PPI) network was performed. Furthermore, the long non-coding RNA (lncRNA)-miRNA-mRNA interactions (competing endogenous RNAs, ceRNAs) were investigated.

**Results:**

A total of 17 downregulated miRNAs were revealed between OA and ctrl groups. These DEMs such as has-miR-1202 were mainly enriched in GO functions like histone acetyltransferase binding and KEGG pathways like cellular senescence. The integrated PPI network analysis showed that has-miR-1202, has-miR-33b-3p, has-miR-940, has-miR-4284, and has-miR-4281 were 5 downregulated miRNAs in this network. Furthermore, the lncRNA-miRNA-mRNA interactions such as KCNQ1OT1-has-miR-1202-ETS1 were revealed in the present ceRNA network.

**Conclusion:**

Key DEMs such as miR-33b-3p, miR-940, and miR-1202 may be involved in OA. miR-1202 may regulate OA development via histone acetyltransferase pathway binding function and cellular senescence pathway. Furthermore, KCNQ1OT1-has-miR-1202-ETS1 might be vital for the process of OA.

## Background

Osteoarthritis (OA) is the wear-and-tear type of arthritis that causes joint pain and stiffness [1]. Worldwide, there are approximately 0.25 billion people suffering with OA [2]. The epidemiology of OA is very complex due to various factors such as genetic and biomechanical components [3], and the molecular mechanisms underlying OA are still not completely understood.

MicroRNAs (miRNAs) are commonly used to identify novel OA genes and their related inflammatory network [4]. A previous study shows that the upregulation of miR-1 controls the development of OA via targeting FZD7 of Wnt/β-catenin signaling [5]. Zhang et al. indicated that miR-320 might target matrix metalloproteinase-13 and further affected the process of OA [6]. Despite miRNAs, long non-coding RNAs (lncRNAs) are correlated with OA and are regulated by OA-associated factors [7, 8]. The previous study shows that lncRNAs take part in many kinds of pathological processes in OA such as extracellular matrix (ECM) [9]. A previous bioinformatics analysis indicates that lncRNAs (such as uc.343) and predicted target homeobox gene C8 (*HOXC8*) are promising therapeutic targets for OA [10]. Actually, the lncRNAs-miRNAs-mRNAs (competing endogenous RNAs, ceRNAs) regulatory network is an important tripartite axis in the regulation of the disease process [11, 12]. Although the identification of molecular factors contributes to the therapeutic interventions in OA [13], the key miRNAs and the possible regulation mechanism of ceRNA in OA are still unclear.

The bioinformatics analysis of gene expression profiles provides new opportunities to uncover the potential OA-related genes such as cathepsin H (*CTSH*) and cathepsin S (*CTSS*) [14–17]. In order to explore the potential differentially expressed miRNAs (DEMs) as a novel breakthrough in the clinical treatment of OA, Ntoumou et al. identified a circulating miRNA signature for OA patients using DEMs analysis and related pathway investigation [18]. They found that miRNAs such as miR-140-3p regulated the metabolic processes of OA patients. However, the integrated regulatory mechanism involving DEMs, mRNAs, and lncRNAs on OA progression is still unclear. Based on the gene expression profile provided by Ntoumou et al., the present bioinformatics analysis revealed the DEMs between OA samples and control samples. Then, the miRNAs-mRNAs relations were explored, followed by the miRNAs-mRNAs regulatory network investigation. The protein-protein interaction (PPI) network was further constructed according to mRNAs in the miRNAs-mRNAs network. Finally, the ceRNA network was constructed and analyzed. The present study aimed to reveal the potential mechanism of OA and provide information about new genes as potential targets for OA treatment.

## Methods

### Data resource and preprocessing

Gene expression profile data in the dataset GSE105027 [18] were downloaded from Gene Expression Omnibus (GEO) database. This dataset was produced on GPL21575 Agilent-070156 Human_miRNA_V21.0_Microarray 046064 platform. A total of 12 serum samples of OA patients (OA group) and 12 serum samples of healthy individuals (ctrl group) were included in the dataset.

The normalization for the downloaded original data was performed using the RAM75 [19, 20] method based on Affy package (version 1.50.0) in Rstudio software (version 1.1.414) [21]. The normalization process in this study included background correction, normalization, and expression quantification. If different probes targeted to the same miRNA (miRNA symbol), the mean value of different probes was considered as the final expression value of this miRNA.

### The investigation for DEMs

The *P* value and fold change (FC) of DEMs between the OA and ctrl group were calculated by Linear Models for Microarray Data (limma) package [22] in R (version 3.32.5) software. Then the *P* value < 0.05 and |log_2_FC| > 1 were selected as the thresholds for the identification of DEMs. The bidirectional hierarchical clustering for DEMs was then performed by pheatmap software (version 1.0.8).

### The miRNA-target gene investigation and network construction

The predicted target genes of miRNAs were investigated using miRWalk 2.0 software (parameters, miRBase database; species, human) [23, 24]. The miRWalk [25], RNA22 [26], miRanda [27], and Targetscan [28] were selected as default databases for interaction scanning. The genes that appeared in all four databases were selected as target genes. Then, we downloaded the target genes of miRNAs which had been already verified in the validated target module of miRWalk 2.0 software. Based on overlapping results of predicted miRNA-target genes and verified miRNA-target genes, the miRNA-mRNAs interactions were finally explored. Furthermore, the miRNA-mRNAs interaction relations were visualized by Cytoscape (version 3.2.1, http://apps.cytoscape.org/) [29].

### Enrichment analysis of the DEGs

The clusterProfiler software [30] is an online tool that provides enrichment analyses including Gene Ontology (GO) and Kyoto Encyclopedia of Genes and Genomes (KEGG). Based on the clusterProfiler software, the GO functional annotation and KEGG pathway enrichment analysis were performed on DEGs. *P* value (the significance threshold of the hypergeometric test) < 0.05 was chosen as the cutoff criterion for present enrichment analysis.

### Construction of PPI network

The PPI research could reveal the interactions of proteins at the molecular level [31]. Search Tool for the Retrieval of Interacting Genes/Proteins (STRING, version 10.0) is a biological database of known and predicted PPI [32]. Using STRING database, PPI was constructed by the target genes that were regulated by miRNAs (required confidence (combined score) > 0.4). Then, the integrated PPI network was further constructed based on the PPI network constructed by target genes regulated by miRNA and miRNA-target interactions, followed by visualization using Cytoscape software. The network topology analyses, including degree centrality (DC) [33], betweenness centrality (BC) [34], and closeness centrality (CC) [35], were performed by the CytoNCA [36] (version 2.1.6; parameter, without weight) to reveal the hub protein [19].

### Construction of ceRNA regulatory network

The miRNA-associated lncRNAs were explored based on the lnCeDB database (http://www.lncrnablog.com/) [37]. The lncRNA-miRNA regulatory relations were constructed using Cytoscape software. Then, based on the lncRNA-miRNA relations and miRNA-DEMs interactions, the lncRNA-miRNA-mRNA (ceRNA) network was further explored. Finally, the ceRNA network was visualized by Cytoscape software.

## Results

### DEMs investigation between the OA group and ctrl group

**Fig. 1:**
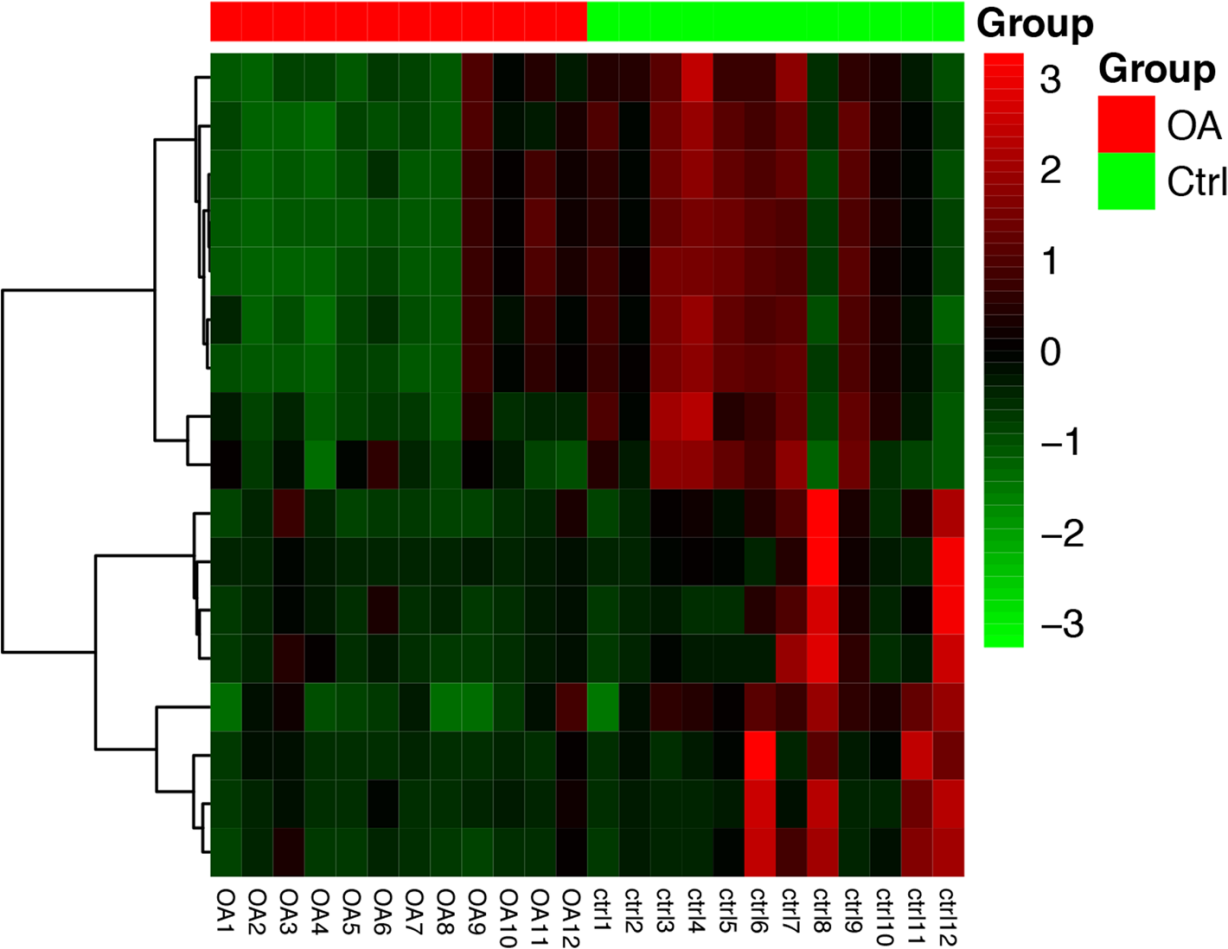
The heat map for differentially expressed miRNAs between the osteoarthritis group and control group. The red block represents the genes in the osteoarthritis group; the green block represents the genes in the control group; and the red color represents upregulation, while green color represents downregulation

With *P* value < 0.05 and |log_2_FC| > 1, a total of 17 downregulated DEMs were obtained between the OA and ctrl groups. However, the result of the upregulated DEMs was negative. The bidirectional hierarchical clustering for DEMs is shown in Fig. 1. The results showed that the expression value of OA samples and ctrl samples could basically separate the two groups.

### The miRNAs-mRNAs interaction analysis

**Fig. 2:**
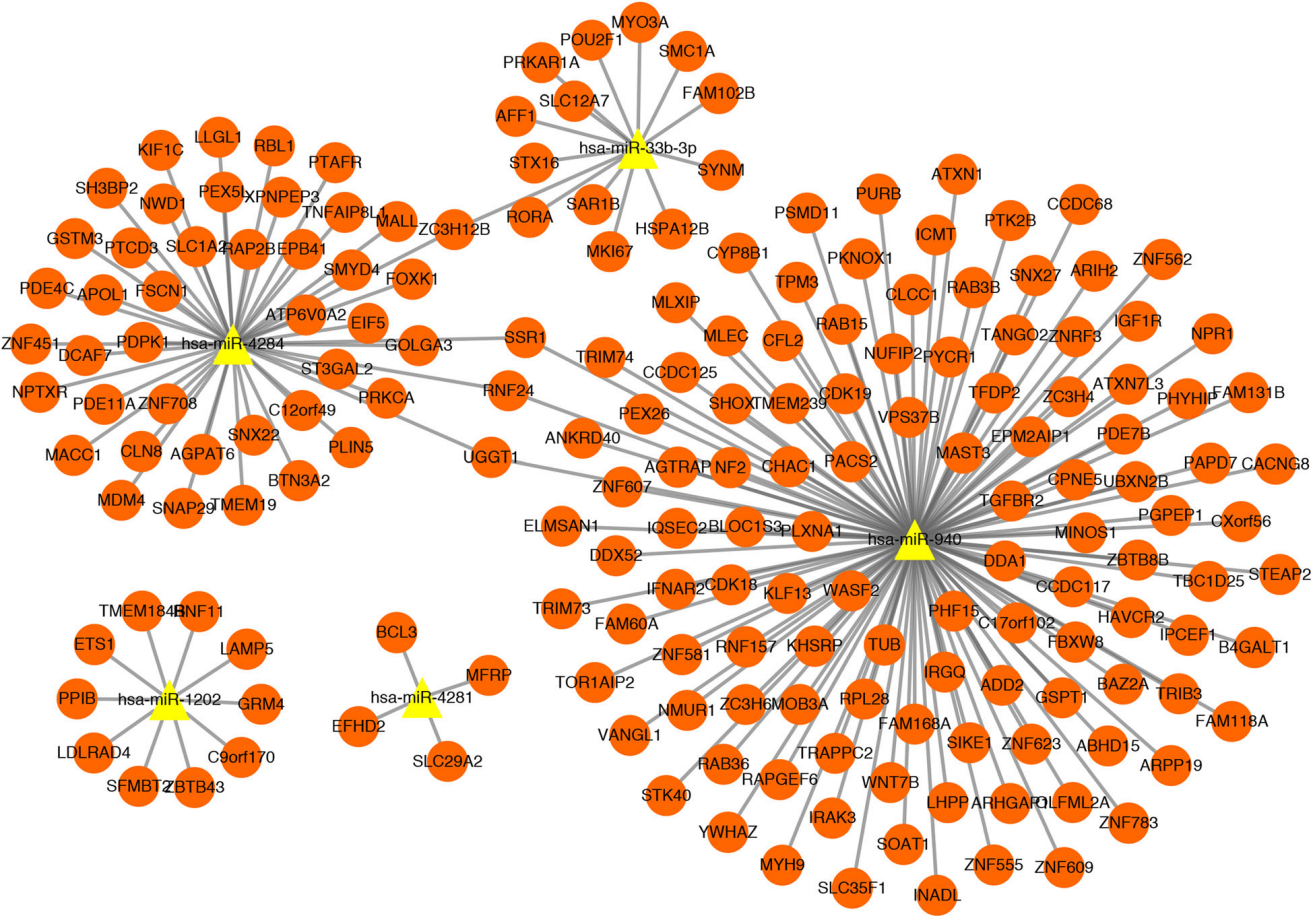
The miRNAs-mRNAs regulatory network. The orange dot represents mRNA, and the yellow triangle represents the downregulated miRNA

A total of 5 downregulated miRNAs (has-miR-1202, has-miR-33b-3p, has-miR-940, has-miR-4284, has-miR-4281) and 185 target mRNAs were screened by miRWalk 2.0 software. Then, the miRNAs-DEMs regulatory network (including a total of 190 nodes and 189 interactions) was constructed (Fig. 2).

### GO and KEGG analysis

The results of the GO function and KEGG pathway enrichment analysis are listed in Tables 1 and 2, respectively. Hsa-miR-1202 was mainly involved in GO functions including histone acetyltransferase binding (GO:0035035; *P* value, 1.28E−02; gene, ETS proto-oncogene 1, transcription factor (*ETS1*)) and glutamate receptor activity (GO:0008066; *P* value, 1.28E−02; gene, Rattus norvegicus glutamate receptor, metabotropic 4 (*GRM4*)). Meanwhile, has-miR-1202 was mainly enriched in pathways like cellular senescence (hsa05211; *P* value, 4.29E−02; gene, *ETS1*) and taste transduction (hsa04742; *P* value, 2.24E−02; gene, *GRM4*).

**Table 1:** The results of GO function analysis for downregulated miRNAs

Cluster	GO	***P*** value	Count	Symbol
hsa-miR-1202	GO:0070412 R-SMAD binding	1.09E−02	1	LDLRAD4
hsa-miR-1202	GO:0008066 glutamate receptor activity	1.28E−02	1	GRM4
hsa-miR-1202	GO:0035035 histone acetyltransferase binding	1.28E−02	1	ETS1
hsa-miR-1202	GO:0070063 RNA polymerase binding	1.75E−02	1	PPIB
hsa-miR-1202	GO:0003755 peptidyl-prolyl cis-trans isomerase activity	2.04E−02	1	PPIB
hsa-miR-1202	GO:0016859 cis-trans isomerase activity	2.13E−02	1	PPIB
hsa-miR-1202	GO:0005518 collagen binding	3.16E−02	1	PPIB
hsa-miR-1202	GO:0046332 SMAD binding	3.30E−02	1	LDLRAD4
hsa-miR-1202	GO:0051082 unfolded protein binding	4.73E−02	1	PPIB
hsa-miR-33b-3p	GO:0003774 motor activity	4.55E−03	2	MYO3A/SMC1A
hsa-miR-33b-3p	GO:0019215 intermediate filament binding	1.01E−02	1	SYNM
hsa-miR-33b-3p	GO:0030898 actin-dependent ATPase activity	1.01E−02	1	MYO3A
hsa-miR-33b-3p	GO:0034236 protein kinase A catalytic subunit binding	1.16E−02	1	PRKAR1A
hsa-miR-33b-3p	GO:0000146 microfilament motor activity	1.70E−02	1	MYO3A
hsa-miR-33b-3p	GO:0001221 transcription cofactor binding	1.70E−02	1	RORA
hsa-miR-33b-3p	GO:0030552 cAMP binding	1.85E−02	1	PRKAR1A
hsa-miR-33b-3p	GO:0030291 protein serine/threonine kinase inhibitor activity	2.23E−02	1	PRKAR1A
hsa-miR-33b-3p	GO:0043531 ADP binding	2.53E−02	1	MYO3A
hsa-miR-33b-3p	GO:0030551 cyclic nucleotide binding	2.76E−02	1	PRKAR1A
hsa-miR-33b-3p	GO:0005484 SNAP receptor activity	2.91E−02	1	STX16
hsa-miR-33b-3p	GO:0008307 structural constituent of muscle	3.06E−02	1	SYNM
hsa-miR-33b-3p	GO:0051018 protein kinase A binding	3.21E−02	1	PRKAR1A
hsa-miR-33b-3p	GO:0032934 sterol binding	3.29E−02	1	RORA
hsa-miR-33b-3p	GO:0004879 RNA polymerase II transcription factor activity, ligand-activated sequence-specific DNA binding	3.74E−02	1	RORA
hsa-miR-33b-3p	GO:0015296 anion:cation symporter activity	3.74E−02	1	SLC12A7
hsa-miR-33b-3p	GO:0098531 transcription factor activity, direct ligand regulated sequence-specific DNA binding	3.74E−02	1	RORA
hsa-miR-33b-3p	GO:0003707 steroid hormone receptor activity	4.41E−02	1	RORA
hsa-miR-33b-3p	GO:0003682 chromatin binding	4.85E−02	2	POU2F1/SMC1A
hsa-miR-4281	GO:0005337 nucleoside transmembrane transporter activity	3.10E−03	1	SLC29A2
hsa-miR-4281	GO:0015932 nucleobase-containing compound transmembrane transporter activity	6.91E−03	1	SLC29A2
hsa-miR-4281	GO:1901505 carbohydrate derivative transporter activity	7.15E−03	1	SLC29A2
hsa-miR-4281	GO:0030674 protein binding, bridging	3.63E−02	1	BCL3
hsa-miR-4281	GO:0060090 binding, bridging	4.00E−02	1	BCL3
hsa-miR-4284	GO:0004114 3′,5′-cyclic-nucleotide phosphodiesterase activity	1.64E−03	2	PDE4C/PDE11A
hsa-miR-4284	GO:0004112 cyclic-nucleotide phosphodiesterase activity	1.78E−03	2	PDE4C/PDE11A

*GO* Gene Ontology; *P* < 0.05 was considered to be significantly different

**Table 2:** The results of KEGG pathway enrichment analysis for down-regulated miRNAs

Cluster	Pathway	***P*** value	Count	Gene
hsa-miR-1202	(hsa05211) Renal cell carcinoma	1.86E−02	1	ETS1
hsa-miR-1202	(hsa04742) Taste transduction	2.24E−02	1	GRM4
hsa-miR-1202	(hsa04724) Glutamatergic synapse	3.06E−02	1	GRM4
hsa-miR-1202	(hsa04072) Phospholipase D signaling pathway	3.92E−02	1	GRM4
hsa-miR-1202	(hsa04218) Cellular senescence	4.29E−02	1	ETS1
hsa-miR-33b-3p	(hsa04966) Collecting duct acid secretion	2.53E−02	1	SLC12A7
hsa-miR-33b-3p	(hsa04710) Circadian rhythm	2.90E−02	1	RORA
hsa-miR-33b-3p	(hsa04130) SNARE interactions in vesicular transport	3.18E−02	1	STX16
hsa-miR-4281	(hsa04668) TNF signaling pathway	1.46E−02	1	BCL3
hsa-miR-4284	(hsa05032) Morphine addiction	2.05E−03	3	PDE4C/PDE11A/PRKCA
hsa-miR-4284	(hsa05143) African trypanosomiasis	4.33E−03	2	APOL1/PRKCA
hsa-miR-4284	(hsa04960) Aldosterone-regulated sodium reabsorption	4.83E−03	2	PDPK1/PRKCA

*KEGG* Kyoto Encyclopedia of Genes and Genomes; *P* < 0.05 was considered to be significantly different.

Hsa-miR-33b-3p was mainly involved in GO functions including motor activity (GO:0003774; *P* value, 4.55E−03; gene, myosin IIIA (*MYO3A*)) and microfilament motor activity (GO:0000146; *P* value, 1.70E−02; gene, *MYO3A*). Meanwhile, hsa-miR-33b-3p was mainly enriched in KEGG pathways like collecting duct acid secretion (hsa04966; *P* value, 2.53E−02; gene, solute carrier family 12 member 7 (*SLC12A7*)) and circadian rhythm (has04710; *P* value, 2.90E−02; gene, retinoid-related orphan receptor alpha (*RORA*)).

Hsa-miR-4281 was mainly involved in GO functions including nucleoside transmembrane transporter activity (GO:0005337; *P* value, 3.10E−03; gene, solute carrier family 29 member 2 (*SLC29A2*)) and nucleobase-containing compound transmembrane transporter activity (GO:0015932; *P* value, 6.91E−03; gene, *SLC29A2*). Meanwhile, hsa-miR-4281 was mainly enriched in KEGG pathways like TNF signaling pathway (hsa04668; *P* value, 1.46E−02; gene, B cell CLL/lymphoma 3 (*BCL3*)).

Hsa-miR-4284 was mainly involved in GO functions including 3′,5′-cyclic-nucleotide phosphodiesterase activity (GO:0004114; *P* value, 1.64E−03; gene, phosphodiesterase 4C (*PDE4C*)) and cyclic-nucleotide phosphodiesterase activity (GO:0004112; *P* value, 1.78E−03; gene, *PDE4C*). Meanwhile, hsa-miR-4284 was mainly enriched in pathways like morphine addiction (hsa05032; *P* value, 2.05E−03; gene, protein kinase C alpha (*PRKCA*)) and African trypanosomiasis (hsa05143; *P* value, 4.33E−03; gene, *PRKCA*).

### PPI network analysis

**Fig. 3:**
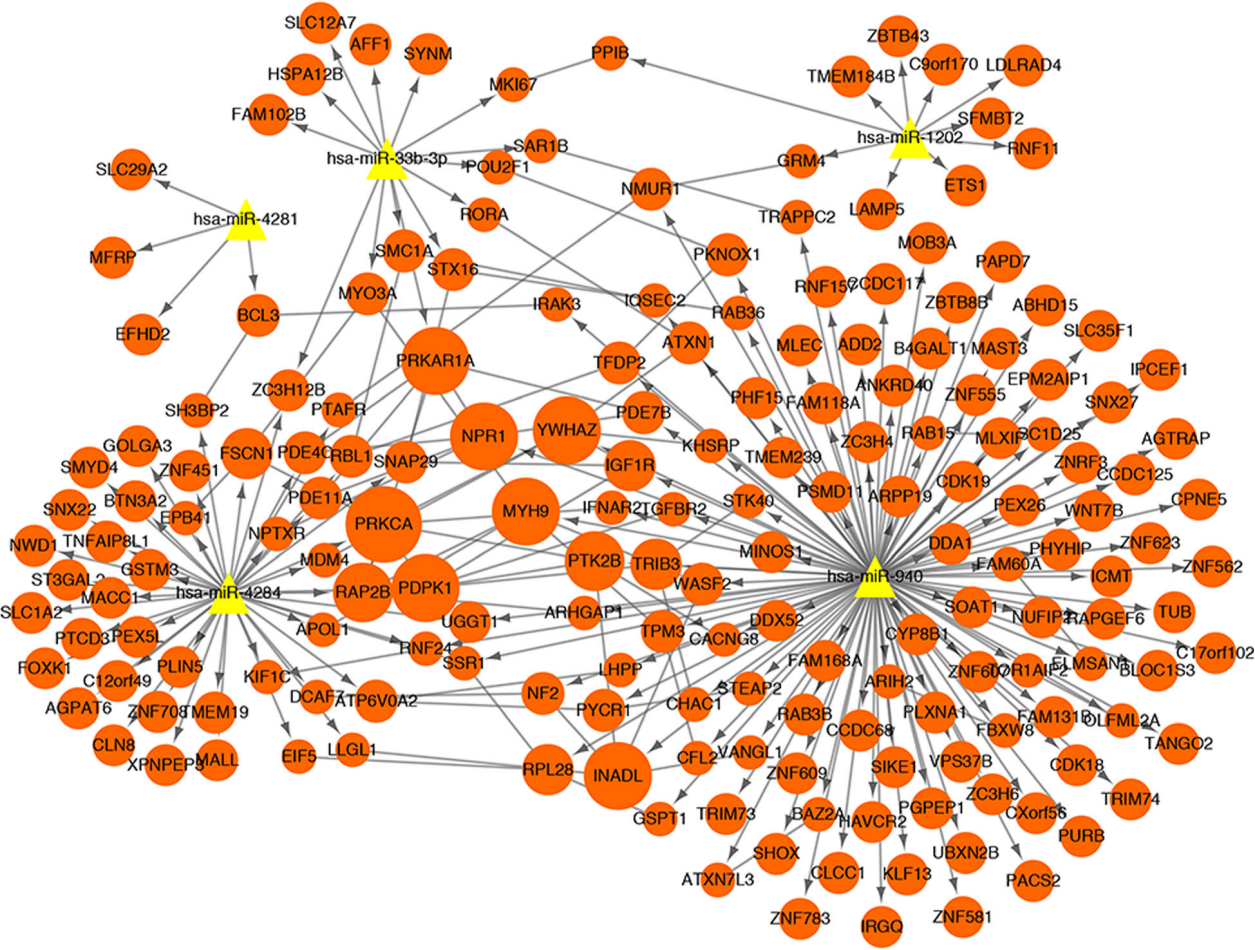
The integrated protein-protein interaction network in present study. The red circle represents the mRNA, the yellow triangle represents the downregulated miRNA, the line without arrow represents the protein-protein interaction relation, and the line with an arrow represents miRNA-mRNA relationship. The dot size represents the correlation degree of node in PPI network; the larger the number of dots; the larger the dots, the greater the genetic correlation degree

The PPI network was constructed with the target genes of DEMs using STRING database. With a combined score of > 0.4, a total of 68 interaction relations and 75 nodes were identified in the PPI network. Based on the topology analysis, the top 5 hub proteins (according to the degree) were PRKCA (DC, 6.0; BC, 743.0; CC, 0.02647585), InaD-like protein (INADL) (DC, 5.0; BC, 319.0; CC, 0.026176158), cAMP-dependent protein kinase type I-alpha regulatory subunit (PRKAR1A) (DC, 5.0; BC, 333.0; CC, 0.026111504), myosin heavy chain type II isoform A (MYH9) (DC, 5.0; BC, 309.0; CC, 0.026222536) and natriuretic peptide receptor A/guanylate cyclase A (NPR1) (DC, 5.0; BC, 77.0; CC, 0.025819957). The integrated PPI network included 190 nodes, 257 relations, and 5 downregulated miRNAs (including has-miR-33b-3p, has-miR-1202, has-miR-4281, has-miR-4284, and has-miR-940) (Fig. 3).

### ceRNA regulatory network investigation

**Fig. 4:**
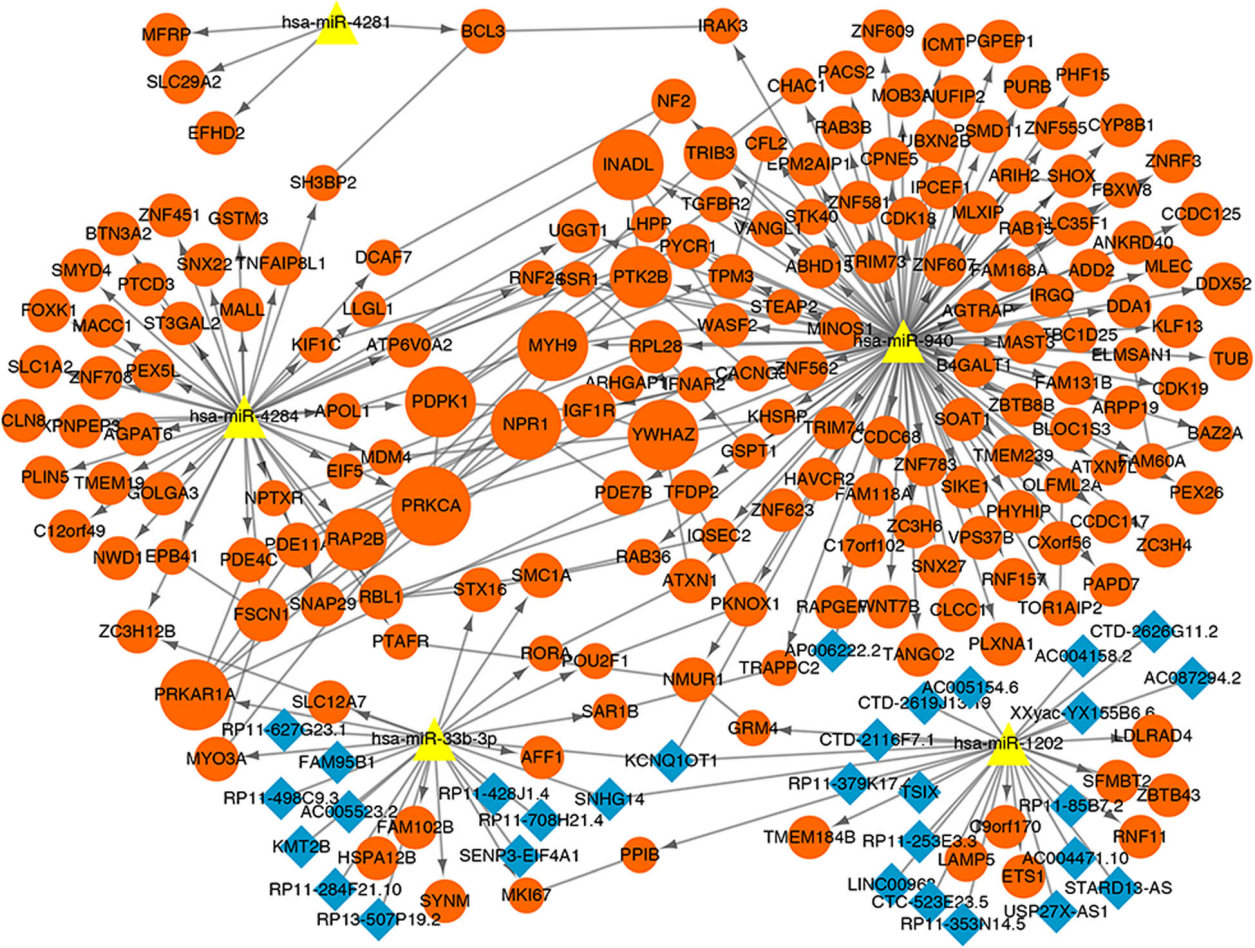
The ceRNA network in this study. The orange dot represents mRNA; the blue diamond represents lncRNA; and the yellow triangle represents the downregulated miRNA. The dot size represents the correlation degree of node in PPI network; the larger the dots, the greater the genetic correlation degree

The lnCeDB database was used to explore the lncRNAs related with 5 miRNAs mentioned above. The results showed that a total of 172 lncRNAs and 215 interactions were investigated in miRNAs-lncRNAs regulatory relations. With the binding-site number > 2, a lncRNA-miRNA regulatory network (including 3 miRNAs and 30 lncRNAs) was constructed using Cytoscape software. Then, based on the lncRNA-miRNA relations and miRNA-DEMs interactions, the lncRNA-miRNA-mRNA interactions (ceRNA) including KCNQ1 overlapping transcript 1 (KCNQ1OT1)-has-miR-1202-ETS1 was further explored. Finally, the ceRNA network was visualized by Cytoscape software (Fig. 4).

## Discussion

OA is the most common form of joint disease [38]. miRNAs are expressed in a different fashion in osteoarthritic relative to nonosteoarthritic cartilage and may be useful for diagnosis or management of OA [33, 34]. In the present study, a total of 17 downregulated miRNAs were revealed between the OA group and ctrl group. These DEMs such as has-miR-1202 were mainly enriched in GO functions like histone acetyltransferase binding and KEGG pathways like cellular senescence. The integrated PPI network analysis showed that has-miR-1202, has-miR-33b-3p, has-miR-940, has-miR-4284, and has-miR-4281 were 5 downregulated miRNAs in this network. Furthermore, the lncRNA-miRNA-mRNA interactions such as KCNQ1OT1-has-miR-1202-ETS1 were revealed in the present ceRNA network.

miRNAs are increasingly implicated in the pathogenesis of complex diseases such as cancer and OA [35, 39]. Based on bioinformatics and proteomic analysis, Dimitrios et al. indicated that the collaborative networks of miRNAs and proteins were associated with OA pathogenesis [40]. In previous studies, serum miRNA array analysis showed that miR-33b-3p may be used as a potential biomarker for OA [41]. Meanwhile, miR-940 is involved in the inflammatory response of chondrocytes in OA [42]. In this study, miR-33b-3p and miR-940 were downregulated in the OA group, suggesting the potential role of them in OA. As a member of the miRNA family, miR-1202 suppresses proliferation and induces endoplasmic reticulum stress in tumor cells, which suggest a potential therapy of miR-1202 in clinical treatment [43]. Despite that, miR-1202 is a primate-specific and brain-enriched miRNA [44]. However, the function of miR-1202 in OA is still unclear. In this study, miR-1202 was an outstanding downregulated miRNA between the OA group and ctrl group, suggesting that miR-1202 might take part in the progression of OA.

In addition, complex interactions existed between miRNAs and their multiple target genes, which may be important in gene regulation and the control of homeostatic pathways in OA [33, 34]. As a target gene of miR-1202, *ETS1* is a member of the ETS family of transcription factors [45]. *ETS1* has been proved to associate with regulation of immune cell function and with an aggressive behavior in tumors [46]. Xu et al. indicated that miR-221-3p modulated *ETS1* expression in synovial fibroblasts of OA patients [47], suggesting the potential role of *ETS1* in OA. Histone acetylation has a close relation with some biological effects such as gene transcription and is catalyzed by histone acetyltransferases [48]. A previous study shows that *ETS1* synergistically activates the gene expression of guanylyl cyclase/natriuretic peptide receptor A via histone acetyltransferase pathway [49]. However, the detailed mechanism of *ETS1* and histone acetyltransferase pathway in OA is unclear. Moreover, *ETS1* can activate the p16INK4a promoter through an ETS-binding site via cellular senescence function [50]. In this study, miR-1202 and *ETS1* were enriched in histone acetyltransferase binding function and cellular senescence pathway. Thus, based on the present study, we speculated that the downregulated miR-1202 might target *EST1* and further affect the OA progression via histone acetyltransferase pathway binding and cellular senescence pathway.

LncRNAs are proved to participate in a variety of biological processes as regulatory molecules [51]. The expression of lncRNAs has recently been reported in tumorigenesis and plays a pivotal role in regulating behavior cell cycle [52]. A previous study shows that lncRNA functions as a ceRNA to promote cartilage degradation in human OA [8]. KCNQ1OT1 is a nuclear transcript found in close proximity to the nucleolus in certain cell types [53]. It involves in certain imprinted gene network that may play a role in various diseases including Beckwith-Wiedemann syndrome and coronary artery lesion [54, 55]. However, the effect of KCNQ1OT1 on OA is still unclear. Our ceRNA network analysis showed that KCNQ1OT1-has-miR-1202-ETS1 was one of the most outstanding ceRNA. Thus, we speculated that the lncRNA KCNQ1OT1 might play an important role in OA progression via sponging has-miR-1202-ETS1 interaction.

However, there were some limitations in this study such as small sample size and lack of verification test, which will introduce false-positive results and consequently influence the reliability of our findings. Thus, a large sample size with a wide verification analysis is needed in further investigation.

In conclusion, the present study showed that downregulated DEMs such as miR-33b-3p, miR-940, and miR-1202 may be involved in OA. miR-1202 might target *EST1* to regulate the activation of histone acetyltransferase pathway binding and cellular senescence pathway and thus further affect OA development. Furthermore, KCNQ1OT1-has-miR-1202-ETS1 might play an important role in the process of OA.

## Data Availability

The datasets used and analyzed during the current study are available from the corresponding author on reasonable request.
